# Trace Amounts of Ranavirus Detected in Common Musk Turtles (*Sternotherus odoratus*) at a Site Where the Pathogen Was Previously Common

**DOI:** 10.3390/ani13182951

**Published:** 2023-09-18

**Authors:** Rachel M. Goodman, Henry R. Carman, R. Paul Mahaffy, Nathan S. Cabrera

**Affiliations:** 1 Department of Biology, Hampden-Sydney College, Hampden-Sydney, VA 23943, USA; cabreran24@hsc.edu; 2 The Watershed Research and Training Center, Hayfork, CA 96041, USA; carmanhenry3@gmail.com; 3 School of Physical Therapy, University of Lynchburg, Lynchburg, VA 24502, USA; paulmahaffy@gmail.com

**Keywords:** reptile, disease, pathogen, iridovirus, wildlife disease, Eastern Painted Turtles, *Chrysemys picta picta*

## Abstract

**Simple Summary:**

Ranaviruses are an important source of disease and mortality for reptiles, amphibians, and fishes around the world. In 2021–2022, we surveyed two species of aquatic turtles at a Virginia site where previous research found ranavirus in turtles and lizards. We used 249 samples of skin collected from turtles to determine whether they carried ranavirus using genetic testing. We found very small amounts of ranavirus DNA in 2.8% of Common Musk Turtles, which have not been previously reported to carry ranavirus. The amounts of ranavirus DNA were so small that we think they were picked up from the environment and were probably not indicative of turtles being infected with the virus. We did not find any ranavirus in Eastern Painted Turtles, which contrasts a 2010 study wherein 23.8% of turtles of this species in the same site carried ranavirus. The amount of ranavirus in our study site, as reflected in the skin samples from the turtles, appears to have dropped dramatically since previous research conducted over a decade ago. Because we only detected ranavirus in 4 out of 249 skin samples, and in only one of 2 years, we emphasize the need for large sample sizes and multiyear sampling to detect ranavirus in wild populations.

**Abstract:**

Ranaviruses are global multi-host pathogens that infect ectothermic vertebrates and cause mass mortality events in some species. In 2021–2022, we surveyed two species of aquatic turtles in a Virginia site where previous research found ranavirus in lizards (*Sceloporus undulatus*) and turtles (*Chrysemys picta picta* and *Terrapene carolina carolina*). We sampled tissues from 206 turtles and tested 249 samples (including recaptures) for ranavirus using qPCR. We detected trace amounts of ranavirus DNA in 2.8% of Common Musk Turtles (*Sternotherus odoratus*). We did not detect the virus in Eastern Painted Turtles (*C. p. picta*). The Ct values from animals carrying ranavirus corresponded to positive controls with a concentration of one copy of ranavirus DNA per microliter and likely reflect DNA in the environment rather than ranavirus infection in turtles. Turtles carrying ranavirus DNA came from only one pond in one year. The amount of ranavirus in our study site, as indicated by tissue samples from turtles, appears to have dropped dramatically since previous research conducted over a decade ago. This study represents the first report of ranavirus detected in *S. odoratus* and contributes to the scarce literature on longitudinal surveys of ranavirus in wild chelonians. We emphasize the need for large sample sizes and multi-year sampling to detect this pathogen in wild populations.

## 1. Introduction

### 1.1. Background on Ranaviruses and Relevant Research in Reptiles

Ranaviruses (family *Iridoviridae*, genus *Ranavirus*) are emerging infectious diseases that have a broad host range, infecting amphibians, fish, and reptiles [1]. They are large, double-stranded viruses that replicate in temperatures of 12–32 °C and can be transmitted via contact and consumption of infected hosts (direct contact) and water, fomites, and sediment (indirect contact) [1,2]. Ranaviruses have caused mass die-off events in amphibians, reptiles, and fish [3,4,5,6,7] and may be transmitted between these taxonomic classes [8,9]. Ranaviruses have been found in at least 175 species across 52 families and reported from across the United States and all continents except Antarctica [6,10]. The majority of surveys and reports of ranavirus occurrence have been conducted in amphibians because of severe negative impacts on some species and the alarming fact that 41% of amphibians are threatened globally [2,6,7,11]. Publications in the Global Ranavirus Reporting System describing ranavirus in wild reptiles number less than 20% of those describing ranavirus in wild amphibians [10]. Reptiles merit increased attention and research with respect to this pathogen, considering that 21% of reptile species are threatened and the proportion increases to 57.9% when considering only turtles [11,12,13].

External symptoms of ranavirosis in chelonians may include nasal and ocular discharge, stomatitis, oral plaques, aural abscesses, conjunctivitis, and subcutaneous edema, particularly in the neck and inguinal region [14,15,16,17]; however, some studies with inoculated juvenile turtles have noted a lack of external symptoms other than lethargy in infected animals [14,18]. Internal symptoms of ranavirosis in turtles may include lesions and necrosis in the upper respiratory tract, gastrointestinal tract, spleen and liver, petechia in the glottis, liver, pancreas, and fat, splenomegaly, gastrointestinal hemorrhage, hepatic lipidosis and sepsis, and inclusion bodies [17,19,20,21]. Deaths attributed to ranavirus are thought to be caused by multiorgan failure [17].

Ranaviruses have been detected in 21 turtle species from seven countries in captivity and the wild; however, research is heavily tilted toward a few species, and most species have not been assessed as carriers of ranavirus in the wild [6,17]. Challenge experiments have been conducted with FV3, the type species of *Ranavirus*, in Red-eared Slider Turtles (*Trachemys scripta elegans*; readily available in the reptile husbandry industry) to examine the influence of herbicide exposure [18] and in this species and Mississippi Map Turtles (*Graptemys pseudogeographica kohnii*), False Map Turtles (*Graptemys pseudogeographica*), and Eastern River Cooters (*Pseudemys concinna concinna*) to examine the influence of temperature [14,15]. In Australia, BIV (Bohle iridovirus isolate; FV3 species) has been detected in wild turtles in northern Queensland [22] and used in challenge experiments to demonstrate pathogenicity in chelonians and influence of temperature and exposure time [23,24]. In North America, FV3 and FV3-like ranaviruses have been reported in the wild in one Gopher Tortoise in Florida (*Gopherus polyphemus*) [25], one Eastern Mud Turtle (*Kinosternon subrubrum*) in South Carolina [26], one Common Snapping Turtle in southern Ontario (*Chelydra serpentina*) [20], Eastern Painted Turtles in Virginia (*Chrysemys picta picta*) [27], a Florida box turtle (*Terrapene carolina bauri*) in Florida [25], and Eastern Box Turtles in Georgia, Pennsylvania, New York, North Carolina, Tennessee, Texas, and Virginia (*Terrapene carolina carolina*) [25,28,29,30]. In *T. c. carolina*, outbreaks have led to population declines of 28–71% over 2–3 years (reviewed in [17]); however, we know little about the population-level dynamics of ranavirus in other turtles due to a lack of regular monitoring or only single individuals presenting symptoms that result in testing for ranavirus. 

### 1.2. Introduction to the Study System and Objectives

This study was intended to follow up on surveys conducted during 2010–2014 [27,29] in a rural college campus in central Virginia wherein ranavirus was found in three species of reptiles. Specifically, FV3-like ranavirus occurred in Eastern Painted Turtles (*C. p. picta*), Eastern Box Turtles (*T. c. carolina*), and Eastern Fence Lizards (*Sceloporus undulatus*) occupying terrestrial and aquatic habitats on campus, with a prevalence of 23.8%, 20.0%, and 36.1%, respectively. No animals in either study, including those carrying ranavirus, displayed any disease symptoms consistent with ranavirosis. However, the surveys were short-term in nature, with most sampling occurring during 8 weeks in one or two summers for each species. The ponds, woods, and walking trails in these studies are frequented by students, faculty, and staff, including regular use for classes and student research projects. In addition, the ponds are frequented by staff for maintenance, and members of the public use them for recreational fishing. Although regular surveys have not been ongoing, there have been no reports of any sick or dead turtles in these areas during the last 15 years despite regular traffic (Goodman, pers. obs., Bill Gillen, pers. comm.). In 2021 and 2022, we surveyed aquatic turtles in the same two ponds from the Goodman et al. [27] study. Specifically, we trapped and collected tissue samples from the two most common aquatic turtle species that are resident on campus, Eastern Painted Turtles (*C. p. picta*) and Common Musk Turtles (*S. odoratus*) and tested them for ranavirus DNA using qPCR. Our objectives were to examine potential differences in ranavirus prevalence between species and years, persistence of ranavirus in individual turtles, and impacts of ranavirus on growth and survival between years.

## 2. Materials and Methods

### 2.1. Capture of Turtles and Collection of Tissue Samples

**Figure 1:**
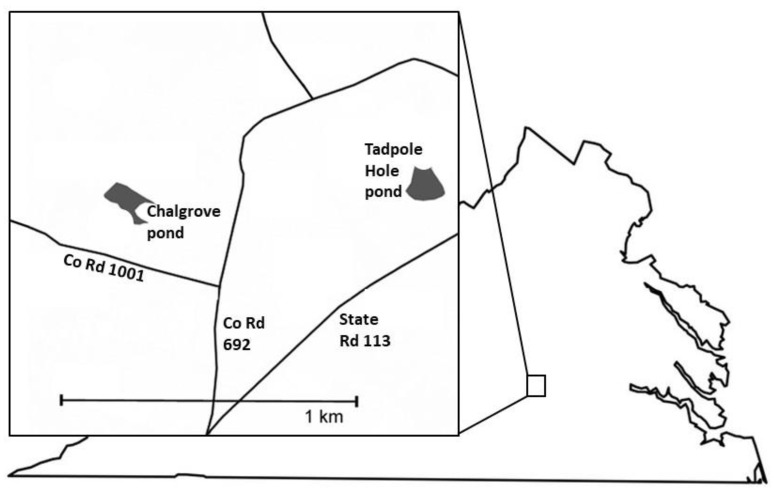
Map of the study area, which is on the campus of Hampden-Sydney College in Prince Edward County, VA, USA. Turtle trapping was conducted at Chalgrove and Tadpole Hole ponds during May–July of 2021 and 2022.

Turtles were captured over 32 trap days during 6 June–23 July of 2021 and 31 trap days during 25 May–7 July of 2022. We used five Promar collapsible crab/fish traps at two ponds (approx. 1 ha each) on the campus of Hampden-Sydney College in Prince Edward County, VA, USA (Figure 1; Chalgrove pond: 37°14′34.27″ N, 78°27′50.04″ W; Tadpole Hole pond: 37°14′42.74″ N, 78°27′10.66″ W). Traps were set 1–2 m from the shore, baited with chicken livers and gizzards, and checked every 12 hours. We alternated between the ponds each week. Sometimes more than one turtle was found in a trap; however, turtles that were later determined to be carrying ranavirus did not originate from the same traps. Turtles were transported in individual plastic containers to the lab, wherein counters were disinfected between collection days. No known ranavirus-positive animals or challenge experiments were present in the lab during the course of this study or for three years prior. We filed notches into marginal scutes with unique codes for individual identification of turtles upon recapture. Oral-cloacal swabs were previously demonstrated to be ineffective at sampling for ranavirus in *C. p. picta* in this population, and tails are cornified in *S. odoratus*, which means tail tips cannot be collected [27]. Therefore, we used sterile biopsy punches to yield a similar amount of tissue as in the previous study and sampled from the side of the body just anterior to the insertion of the hind limb. First, we applied Orasol Anesthetic Gel (20% benzocaine) to the skin in this area, waited three minutes, and then wiped it dry with sterile gauze. We then used Integra Miltex disposable 4 mm biopsy punches and a sterile scalpel, if necessary, to remove the surface dermal layer. Next, we applied 10% povidone iodine to the area, dabbed the area dry with sterile gauze, and applied 3M Vetbond tissue adhesive to seal the wound. Recaptured turtles within a season showed no signs of wound infection, and the site of tissue collection was barely detectable for turtles captured in the following year. Tissue samples were collected again in 2022 from turtles that were captured in 2021 (specified as recaptures in this study), but not from turtles that were caught more than once in the same year.

In the summers of 2021 and 2022, we caught and sampled tissues from 81 individuals of *C. p. picta*. In 2022, we recaptured and sampled tissues from 24 of those turtles captured in 2021. Between both years, we caught 125 individuals of *S. odoratus*. In 2022, we recaptured and sampled tissues from 19 of those turtles captured in 2021. Between both years and both species, we caught and sampled 206 turtles. Including recaptures, we tested 249 tissue samples from turtles (*n* = 99 in 2021; *n* = 150 in 2022).

Turtles were checked 10 min after processing to ensure the integrity of the wound sealing method, and they were released at their trapping site within 24 h of capture. They were handled with nitrile gloves from the time of trapping until release. Gloves were changed, and all lab equipment was disinfected with 70% ethanol or 1% Nolvasan (Zoetis Inc., Kalamazoo, MI, USA) between contact with different individuals, with a final water rinse in the case of Nolvasan [31]. Forceps were soaked in water, scrubbed using a soapy brush, rinsed and drained, and then disinfected. Turtle traps were disinfected between uses in different ponds with 1% Nolvasan. Before trap disinfection, mud or debris was removed if present, then rinsed, and then the disinfecting agent was applied and allowed to sit for at least 1 min.

### 2.2. DNA Extraction and Genetic Testing for Presence of Virus 

Upon collection, tissue samples were stored without preservative or buffer (as in previous studies [27,29]) at −80 °C until DNA extraction with DNeasy Blood and Tissue kits (Qiagen, Valencia, CA, USA) followed by quantification of DNA concentrations with an Epoch spectrophotometer (Biotek). We tested for ranavirus using 75 ng of sample DNA in quantitative PCR targeting a 70 base pair region of the MCP gene that detects FV3 and FV3-like ranaviruses, following Gray et al. [32]. As in the previous Goodman et al. study [29], samples were run twice on a StepOne Real-Time PCR machine (Applied Biosystems). Water and serial dilutions of ranavirus gblocks (1 to 10^6^ copies/μL) were used as negative and positive controls, respectively. We initially ran all samples twice, and any sample that resulted in any numeric Ct (cycle threshold level) value was run two more times. Due to our initial results, we also extracted DNA and ran qPCR on samples of liver and kidney tissue from four ranavirus-infected Wood Frogs (*Lithobates sylvaticus*) from another lab to verify that our positive controls were working properly. 

## 3. Results

Only four tissue samples yielded Ct values consistent with trace amounts of ranavirus DNA, based on the criteria that follow, and these all came from *S. odoratus* turtles in Chalgrove pond in 2022 (Table 1). One of these turtles was a recapture, and the associated tissue sample from 2021 did not contain ranavirus DNA. The four aforementioned samples yielded Ct values on at least three out of four qPCR runs, and, on at least one run, they had a Ct value corresponding to a concentration of one copy of ranavirus DNA per μL based on the lowest serial dilution of our positive controls (gblocks). No Ct value for any turtle sample approached the next serial dilution, which contained 10 copies of DNA per μL. For comparison, DNA samples from the four ranavirus-infected frogs (additional positive controls) yielded Ct values corresponding to concentrations of 1000–10,000 copies of ranavirus DNA per μL.

The detection rate for ranavirus in *C. p. picta* and *S. odoratus* (both ponds combined) was 0.0% (95% confidence interval (CI) = 0.0–3.5%, *n* = 105) and 2.8%, respectively (95% CI = 0.8–7.0%, *n* = 144).

## 4. Discussion

### 4.1. Presence of Ranavirus in One Turtle Species at a Very Low Level

Our study represents the first report of ranavirus in *S. odoratus*, adding this species to a short list of five other North American chelonians known to carry ranavirus (reviewed in the Introduction). To our knowledge, there have been no previous surveys of ranavirus in *S. odoratus*, other than the previous Goodman et al. study [27]. They used both oral-cloacal swabs and tissue samples (tail tips) to test for ranavirus; however, the less invasive swab method did not yield any positive test results despite several positive tests with tissue samples in *C. p. picta*. Only oral-cloacal swabs were used in *S. odoratus* because they have cornified tail tips and no ranavirus was detected in that species. However, that result was not considered reliable due to the lack of tissue sampling. Therefore, we cannot directly compare our detection rate for ranavirus to that of the same species sampled in 2010. Using more than one type of sample (oral/cloacal swabs, blood, tissues) has been recommended to increase diagnostic sensitivity for ranavirus [17]; however, the cost of taking more sample types has to be weighed against the cost of sampling more individuals or more time periods when financial resources for genetic testing are limited.

Our original goals with respect to turtle sampling were to examine ranavirus prevalence and persistence between years, differences between species, and impacts on growth and survival. These topics could not be investigated because of the scarcity of ranavirus in our study site. In 2010 and 2013–2014, a substantial portion of *C. p. picta* and *T. c. carolina* carried ranavirus on our college campus (prevalence of 23.8% and 20.0%, respectively). In this current study, we only detected ranavirus in 2.8% of *S. odoratus* and 0% of *C. p. picta* captured during 2021–2022. For ranavirus, the background prevalence is typically less than 5% and a prevalence over 40% may suggest an outbreak [33]; however, we note that these recommendations are mostly based on amphibian studies because surveys of ranavirus in wild reptiles are limited. In addition to having a low detection rate for ranavirus, we found only trace amounts of viral DNA compared to previous research and internal tissues from ranavirus-infected frogs donated by another lab. Combined, these findings lead us to believe that we may have detected ranavirus DNA from the environment carried on the bodies of the turtles, rather than DNA resulting from ranavirus infection in turtles. Unfortunately, we did not collect water or substrate samples at the time of turtle trapping, which would potentially allow us to compare environmental DNA (eDNA) concentrations from abiotic samples to our tissue samples. Future studies could implement this sampling in addition to or in place of collecting animals since testing eDNA in water samples can be a viable alternative to sampling animal tissues for ranavirus detection [34,35,36]. However, environmental sampling would not shed light on host species occurrence, and we lack this information for many species of turtles. Also, note that specialized methods and equipment are required for obtaining eDNA samples, and two dedicated lab spaces are required for DNA extraction and qPCR, with more stringent specifications and technology than those normally used for testing tissues [34,35,36].

### 4.2. Comparisons of Tissue Samples and Genetic Methods with Previous Studies in This System

In this study, we used a biopsy of tissue collected near the insertion of the hind limb so that we could sample from both *C. p. picta* and *S. odoratus* since the latter species presents a cornified tail tip. While this change in tissue sampling between studies may have impacted our ability to detect ranavirus, we cannot imagine a biologically plausible scenario whereby a ranavirus infection would present substantial amounts of DNA in the tail tip but not in the skin on the torso of a turtle. We consider it more likely that the amount of ranavirus has dropped dramatically in our study site and is likely no longer residing in turtles in our study site in appreciable quantities. Ranavirus was detected in the tissues of one species in one pond in 2022 and not at all in 99 turtles in both ponds in 2021. We barely detected ranavirus in this system despite having a greater sample size than in our previous studies: 206 turtles in the current study (*C. p. picta* and *S. odoratus*), versus only 30 *T. c. carolina* from our campus woods in 2013–2014 and 64 turtles (*C. p. picta* and *S. odoratus*) from the same ponds as our current study during 2010 [27,29].

In the previous Goodman et al. study [29], turtles were considered positive for ranavirus if tissue samples yielded Ct values <30 for both runs. At that time, our lab used the same PCR machine and primers but different positive controls, specifically, cultured ranavirus and tissue from experimentally infected animals. At that time, we only used a Ct value cut-off for positive versus negative animals and did not perform dilutions to the low levels of ranavirus DNA that would allow us to detect trace quantities as in the current study. The extremely low concentrations of ranavirus DNA suggested by qPCR for four turtles in the current study would not be considered positive in the 2013–2014 survey when our frame of reference was shaped by a substantial prevalence of ranavirus in *T. c. carolina* (20.0%) and *S. undulatus* (36.1%) [29] and a higher viral quantity as indicated by Ct values of 20–30 (Goodman, unpub. data). In the 2010 turtle survey, ranavirus was detected via conventional PCR and gel electrophoresis, so the Ct values are not available for comparison with the current study.

### 4.3. Implications of This Study for Surveillance of Ranavirus in Wild Populations 

Our results highlight the importance of large sample sizes and repeated sampling to detect ranavirus since prevalence can vary dramatically between seasons and years. Because our research period was limited to summers (in this and previous studies), we have not explored seasonal variation in ranavirus prevalence in our study site. Opportunistic samples from other studies or efforts, such as wildlife rehabilitation centers, provide an important opportunity to survey ranavirus using a nondestructive approach that minimizes stress to animals [37]; however, detection power may be limited when the virus has waned in an ecosystem and samples are limited within species and seasons. We find it noteworthy that we did not detect any ranavirus DNA in 105 tissue samples from 81 individuals of *C. p. picta* collected between 2021 and 2022, where it was commonly found (prevalence 23.8%) and, therefore, easily detected among a sample of only 42 individuals in 2010 [27].

This study only detected ranavirus DNA in *S. odoratus* and not *C. p. picta*, perhaps in part because of the difference in sample size (144 versus 105, respectively), but we suggest that behavioral differences may also play a part. Both species are opportunistic feeders and likely have similar diets in our ponds, including aquatic plants, insects, small fish, and crustaceans [38]. However, whereas we constantly see numerous *C. p. picta* basking on submerged logs and debris in our campus ponds, we almost never see *S. odoratus* basking (including dedicated regular observations of basking using trail cameras for an unrelated study) [39]. In fact, we almost never see them outside of trapping periods despite their greater numbers when captured. Ranaviruses vary by species in optimal thermal range but, generally, they replicate best in 24–28 °C [6,40]. In one population of *C. p. picta* in North Carolina that has a similar climate to our study site in the summer, turtles exhibited mean weekly maximum shell temperatures over 30 °C during June and July [41]. Unfortunately, we are unaware of comparable thermoregulatory studies for *S. odoratus* in the mid-Atlantic or southern portion of its range. We suggest that *S. odoratus* may maintain lower external body temperatures and more constant contact of skin with water due to its lack of basking at our study site. These factors may explain the presence of ranavirus DNA on the skin surface in *S. odoratus* and its absence from the skin surface in *C. p. picta*; however, this hypothesis is speculative and needs to be tested. It would be interesting to compare the prevalence of ranavirus in these two species in the previous study in 2010 [27] when ranavirus appeared to be infecting turtles asymptomatically rather than just present in trace environmental quantities. Unfortunately, that study only captured 22 individuals of *S. odoratus* and only used swab samples, which were shown to be ineffective for detecting ranavirus.

Sampling in the same location over several seasons and years is recommended to understand the impact and dynamics of ranavirus on populations [33]. However, few studies examine community-level dynamics of ranavirus over years, and those that do have focused on amphibians [42,43,44,45]. Bienentreu et al. [43] found that the prevalence of ranavirus varied between species in the same field site and season (0%, 5%, and 43% for three species) and between consecutive years within the same site (0% and 0% versus 13% and 25% for two species). Todd-Thompson [45] only detected ranavirus in salamanders during one 3-week period despite sampling once or twice per month for seven months in a site. The cost of genetic testing in a long-term longitudinal surveillance program is often prohibitive, especially for multiple species, and, therefore, rarely executed [33,46]. However, our study underlines the importance of repeated sampling for detecting, much less investigating the impacts of ranavirus, since prevalence can vary dramatically between years. Reptiles in particular are understudied in this regard, so longitudinal multi-species surveys are needed to understand how ranavirus cycles within and impacts these populations.

### 4.4. Considerations with Respect to Management Practices in Our Study Site

In a previous study, ranavirus was detected in both campus ponds in 2010 [27], whereas, in the current study, it was only detected in Chalgrove but not Tadpole Hole, and only in 2022. The college has not conducted any fish stocking of the ponds in the last 15 years (Bill Gillen, pers. comm.). However, two nonresident turtles have been captured in the ponds during the course of research (R. Goodman, pers. obs.; Eastern River Cooter, *Pseudemys concinna concinna*, Chalgrove, 2010; Red-eared Slider Turtle, *Trachemys scripta elegans*, Tadpole Hole, 2021) indicating that students or local residents may occasionally release animals into the ponds. Interestingly, the college started a contract for regular herbicide application in its ponds to control algae and invasive macrophytes around 2010–2011; however, the ponds in this study were not included until 2012 at the earliest (Nathan Kuhn, pers. comm.). Since then, Tadpole Hole has been treated in most years with copper carbonate and/or Flumioxazin, and, in the last 3–4 years, it has received 1–2 applications of glyphosate annually (either Roundup^®^ Custom, MONSANTO Company, St. Louis, MO, USA or Aquaneat^®^, Riverdale Chemical, Chicago Heights, IL, USA). In contrast, Chalgrove pond requires almost no chemical treatment and the applier recalls only limited treatment of copper carbonate, with the most recent application occurring at least 5 years ago. We do not know if the presence of ranavirus in Chalgrove but not Tadpole Hole may be related to differences in chemical management or other factors. We were unable to find any literature on interactions between ranavirus and Flumioxazin or copper carbonate. Experiments examining the impacts of glyphosate on ranavirus exposure have found a negative impact [47] or no impact on morbidity and mortality on salamanders [48] and no effect on turtles [18]. Forson and Storfer [49] showed that moderate concentrations of another herbicide, atrazine, compromised viral efficacy and reduced impacts on host survival and development in an experimental setting, whereas higher concentrations of the herbicide increased ranavirus-associated mortality, presumably due to impaired immune response in the host. Most research examining interactions between ranavirus and pesticides, including the aforementioned studies and others, focuses on host infection and survival rates in the face of a substantial load of the pathogen and the chemical [50,51,52]. We therefore know little about the direct impacts of pesticide applications on persistence and viability of ranavirus, especially in systems where it occurs at very low levels, as in the current study.

## 5. Conclusions

Ranaviruses are an emerging threat to ectothermic vertebrates, and surveillance in wild reptiles in particular is lacking. The majority of chelonian species in the world are threatened, and the prevalence and disease burden of ranavirus is unknown for most turtle species. This study surveyed aquatic turtles in a study site where ranavirus was previously common in two turtle species, *C. p. pita* and *T. c. carolina*. We detected only trace amounts of the pathogen, with very low detection rates in one species, *S. odoratus*, in only one year of this study. No ranavirus was found in either pond in 2021, despite testing tissue samples from 99 turtles from two species collected that year. No ranavirus was detected in *C. p. pita* in this study among 105 samples, in contrast to ranavirus prevalence of 23.8% found in 42 turtles surveyed in 2010. Our study represents the first report of ranavirus in *S. odoratus*, which is one of only six species for which this pathogen has been detected in North America. The fact that only four out of 249 samples contained ranavirus demonstrates the intensive sampling effort that may be needed to detect this pathogen when it occurs at low levels in a system. That we only detected ranavirus in one year but not the other reinforces the need for sampling over multiple seasons and years. We encourage continued monitoring and surveillance of ranavirus in turtles, which are a unique and highly threatened taxon.

## Figures and Tables

**Table 1:** Two turtle species sampled for ranavirus during a 2021–2022 survey using traps in two ponds on a college campus in central Virginia. “Pos” means positive for trace amounts of ranavirus DNA (see text for details), “Neg” means negative, and “Tot” means total.

	Chalgrove			Tadpole Hole			Both Ponds Combined			
	Pos	Neg	Tot	Pos	Neg	Tot	Pos	Neg	Tot	Detection Rate
*Chrysemys picta picta*										
First captures only	0	41	41	0	40	40	0	81	81	0.0%
First captures + recaptures	0	57	57	0	48	48	0	105	105	0.0%
*Sternotherus odoratus*										
First captures only	3	83	86	0	39	39	3	122	125	2.4%
First captures + recaptures	4	95	99	0	45	45	4	140	144	2.8%

## Data Availability

Data is available upon request.
